# Microvascular anastomosis in a challenging setting using a 4 K three-dimensional exoscope compared with a conventional microscope: An *in vivo* animal study

**DOI:** 10.3389/fsurg.2022.1021098

**Published:** 2022-10-20

**Authors:** Zhiping Zhang, Yao Feng, Xia Lu, Bin Yang, Hongqi Zhang, Yan Ma

**Affiliations:** ^1^ Department of Neurosurgery, Xuanwu Hospital, Capital Medical University, Beijing, China; ^2^ Yasargil Microsurgical Training Center, Beijing, China; ^3^China International Neuroscience Institute, Xuanwu Hospital, Capital Medical University, Beijing, China

**Keywords:** 3D exoscope, microvascular anastomosis, animal study, neurosurgery, microscope

## Abstract

**Background:**

Three-dimensional (3D) exoscope systems have been developed and are reported to be adequate alternatives to the conventional microscope. This study aimed to evaluate the feasibility and effectivity of microvascular anastomosis using a 4 K 3D exoscope in an *in vivo* animal study.

**Methods:**

The abdominal aortas of mice were selected as the target vessels for comparing the outcomes of microvascular anastomosis for both the conventional microscope and 3D exoscope. We recorded the vessel separation, temporary occlusion, and total procedure durations. Local conditions at the sutures were also recorded. Typical histopathological images were presented, and the patency of anastomotic vessels within 5 and 30 min were evaluated. All procedures included both superficial and deep anastomosis.

**Results:**

Sixty mice were included in the analysis; the weight and vascular diameter were 38.5 ± 5.8 g and 0.77 ± 0.06 mm, respectively, and around 8 stiches were required. Regarding feasibility, vessel separation duration, temporary occlusion duration, total procedure duration, blood leak, and number of vascular folds between stiches, the results were comparable between the two types of microscopes. The feasibility of anastomosis was also confirmed by pathology. Regarding effectiveness, anastomotic vascular patency at 5 and 30 min were similar for both microscopes. Even in the more difficult scenario of deep anastomosis, the results were comparable.

**Conclusions:**

In a challenging experimental setting, comparable outcomes of microvascular anastomosis were observed for the conventional microscope and 3D exoscope in these animal experiments. Therefore, *in vivo* microvascular anastomosis is feasible and effective using a 3D exoscope.

## Introduction

Cerebral bypass surgery, which relies on micromanipulation, is one of the most technically nuanced of neurosurgical procedures. With the introduction of the surgical microscope in the 1960s, the safety and efficiency of microsurgical intervention has improved due to the development of optical magnification and illumination of the surgical field (1). However, there are some limitations mainly related to ergonomics, visualization, and training for the conventional microscope (2). Detailly, the surgeon is often forced into uncomfortable positions owing to the ocular-dependent visualization when using a conventional microscope. Moreover, the surgical field of view is limited such that neither the assistant nor the visitor can gain a sufficient sense of participation.

In recent years, three-dimensional (3D) exoscope systems have been developed and are reported to be adequate alternatives to the conventional microscope (3). Exoscopes, a new series of optical devices characterized by a small orientable camera equipped with a slight pneumatic arm assembled on a portable base, offer a telescopic vision of the surgical field (4). The devices possess wide operative fields and focal distances long enough to allow nonobstructive positioning, and are easily maneuverable to simultaneously optimize operative angles and surgeon ergonomics. The entire surgical team also shares the same view, facilitating operative workflow and trainee education (5).

In a study by Hafez et al. (6), 1-mm chicken wing vessels were used for either exoscopic or microscopic bypass procedures. They found that both methods produced equally satisfactory results in experimental bypass procedures, while the exoscope offered better visualization. Hafez et al. (7) conducted another study to compare differences between the operating microscope and exoscope for end-to-side bypass procedures at a depth of 9 cm. The results demonstrated that these two devices had comparable procedural quality, and the authors believed that the digital 3D exoscope might become the main operative visualization system in microneurosurgery. However, their extperiments were conducted *in vitro*, which cannot accurately simulate the complex situation *in vivo*. Additionally, although several researchers (2, 5, 8, 9) reported their experience regarding exoscopes applied in clinical cases, the sample size was small; therefore, animal experiments are needed to illustrate the effect of 3D exoscopes applied for microsurgical intervention.

In this study, we aimed to compare the outcomes of microvascular anastomosis using a conventional microscope and 3D exoscope in a challenging setting based on *in vivo* animal experiments.

## Materials and methods

### Animals

Male mice weighing 30–50 g were purchased from Beijing Vital River Laboratory Animal Technology Company (Beijing, China). Experiments were performed under a project license (No.: XW-20201226-1) granted by the institutional ethics committee of Xuanwu Hospital, Capital Medical University, in compliance with the National Institutes of Health Guide for the care and use of animals. All mice were provided with free access to food and water and were allowed at least 2 days to adapt to new the environment without surgery.

### Microvascular anastomosis

The mice were intraperitoneally anesthetized using 50 mg/kg of 1% phenobarbital sodium and were then fixed on the operating table in the supine position. After sterilization with aner's iodine, a middle abdominal incision, approximately 3–3.5 cm in length, was made. The inferior vena cava and abdominal aorta were exposed after retracting the subcutaneous fat and bowel around the perimeter using retractors, the tissue was dissected layer by layer, and the abdominal aorta was dissociated to approximately 7–8 mm in length. The abdominal aorta was temporally blocked with a microclip (Shanghai Medical Instruments Co., Ltd., Shanghai, China) and cut at the midpoint, creating an end-to-end anastomosis. Usually, 7–8 stitches are sutured, and before the last stitch is knotted, heparin saline (250 U/ml) is injected into the anastomosis site with a syringe needle. After the anastomosis was completed, the microclip was loosened; a small cotton pad was used to stop the bleeding by gently applying pressure, and a vascular patency test was performed to check the patency of the blood vessels (10). Mice were euthanized after 30 min of routine observation to analyze vascular patency and perform sodium fluorescein angiography. We then harvested the anastomosed vessel to visualize the folds between stitches, and the target vessel was stored in 10% formalin for further pathological examination. During the procedure, we recorded the vessel separation duration (time from exposure to complete separation), temporary occlusion duration (time from clipping to opening), total procedure duration (time from incising the skin to opening the microclip), and blood leakage at the sutures. All procedures were performed by ZZ and XL.

### 3D exoscope

The 3D exoscope (HawkSight; Mitaka Kohki Co., Ltd., Tokyo, Japan) used a 4-chip CCD 4 K image acquisition and dual fluorescence system (colored indocyanine green fluorescence and yellow fluorescence, based on yellow fluorescence). The video information is collected through the left and right fields of view to simulate the viewing angle of the human eye, and the high-resolution 4 K 3D display is used to present intraoperative images. A comparison between the 3D exoscope and conventional microscope is presented in Table 1.

**Table 1:** Comparison of technical specifications between the 3D exoscope and conventional microscope.

Characteristics	3D exoscope	Conventional microscope
Model	HawkSight	Pico 100
Light source	LED	Halogen light source
Magnification	5–100×	3.4–21.5×
Fluorescence detection	Yes	No
Field of view	20–155 mm	8–61 mm
Displayed video image	3D 4 K	Full-HD
Working distance	200–1,000 mm	250 mm
Movement by controller	Possible	Possible

3D, three-dimensional; LED, light emitting diode; HD, high definition.

### Experimental grouping

**Figure 1:**
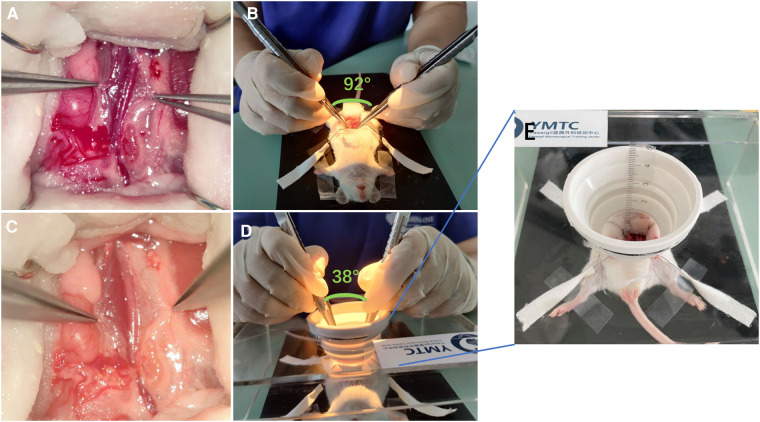
Two scenes of vascular anastomosis with different levels of difficulty. (**A,B**) Superficial anastomosis; the angle between the hands during operation was 38°. (**C,D**) Deep anastomosis; the angle between the hands during operation was 82°. (**E**) Enlarged view of the deep anastomosis device with a depth of about 5 cm.

In this animal experiment, two scenarios (superficial anastomosis and deep anastomosis) were designed. For deep anastomosis, operators were required to perform vascular anastomosis within a 5 cm depth and a exposed field diameter limited to 3 cm Additionally, the two microscopes were simultaneously applied in both scenarios (Figure 1). And animals were randomized by random numbers with the help of BY who was blinded with the procedures.

### Histopathological analysis

The vessel specimens were fixed in 10% neutral-buffered formalin, dehydrated in alcohol, and embedded in paraffin. Samples were sectioned at a thickness of 5 μm for hematoxylin-eosin (HE) staining, and a typical suture cross-section was presented. The pathology analysis was done by YF who was blind to the group.

### Outcome analysis

We recorded the vessel separation, temporary occlusion, and total procedure durations throughout the vascular anastomosis procedure. The results were divided into four groups: (1) superficial anastomosis with the conventional microscope; (2) superficial anastomosis with the 3D exoscope; (3) deep anastomosis with the conventional microscope; and (4) deep anastomosis with the 3D exoscope. No less than 12 in each group. Blood leak was divided into three grades: grade 1, no bleeding was observed, without any additional action after releasing the clip; grade 2, bleeding could be stopped with a piece of cotton pad after loosening the clip; grade 3, bleeding was severe after releasing the clip and required several cotton pads or re-suturing to stop. The evaluation of these outcomes was done by YM and HZ.

### Statistical analysis

Statistical analysis was performed using IBM SPSS Version 23.0 (IBM Corporation, Armonk, New York, USA). Continuous variables are expressed as mean values with standard deviation (SD), while categorical data are expressed as percentages. Comparisons between the conventional microscope and 3D exoscope were performed using Student's *t*-test, the Mann–Whitney *U* test, or the χ^2^ test. Odds ratios (ORs) or *β* were calculated with their 95% confidence intervals (CIs). A *P* value <0.05 was considered statistically significant.

## Results

Experiments were performed on 63 mice in this study. Three mice died during the perioperative period, including one death due to anesthesia overdose and two deaths due to the surgical procedure. Consequently, 60 mice were included in our analysis. Targeted, anastomosed vessels were smaller than 1 mm, and on average, almost eight stiches were required to complete the end-to-end anastomosis. The detailed characteristics of the different experimental groups are presented in Table 2.

**Table 2:** Characteristics of different experimental groups.

Characteristics	All	Superficial anastomosis		ORs or *β* [95%CI]	*P*+value	Deep anastomosis		ORs or *β* [95%CI]	*P*+value
	(*n* = 60)	Microscope (*n* = 12)	3D Exoscope (*n* = 18)			Microscope (*n* = 14)	3D Exoscope (*n* = 16)		
Animal baseline									
Weight, g	38.5 ± 5.8	38.6 ± 5.2	37.6 ± 4.1	1.052 [0.888–1.245]	0.573	36.1 ± 5.6	41.7 ± 6.9	0.866 [0.758–0.989]	0.021
Vascular diameter, mm	0.77 ± 0.06	0.77 ± 0.07	0.77 ± 0.05	NA	0.851	0.79 ± 0.05	0.77 ± 0.07	NA	0.434
Stiches, median (IQR)	8 (7,8)	8 (7,8)	8 (7,8)	0.909 [0.350–2.360]	0.699	8 (7,8)	8 (7,8)	0.754 [0.255–2.232]	0.638
Feasibility									
Vessel separation duration, min	19.09 ± 6.56	17.78 ± 7.00	15.93 ± 5.16	1.057 [0.930–1.200]	0.409	23.26 ± 6.50	19.98 ± 6.03	1.092 [0.965–1.236]	0.163
Temporary occlusion duration, min	30.62 ± 8.77	28.75 ± 8.21	29.26 ± 8.76	0.993 [0.908–1.085]	0.874	31.29 ± 7.82	32.99 ± 10.07	0.978 [0.901–1.062]	0.614
Total procedure duration, min	55.94 ± 13.27	52.14 ± 14.32	55.33 ± 13.12	0.982 [0.928–1.038]	0.534	58.89 ± 12.39	56.88 ± 13.90	1.012 [0.957–1.071]	0.681
Blood leak grade									
1	34 (56.7%)	5 (41.7%)	10 (55.6%)	1.301 [0.355–4.775]	0.466	10 (71.4%)	9 (56.3%)	1.944 [0.424–8.919]	0.389
2	25 (41.7%)	7 (58.3)	7 (38.9%)			4 (28.6%)	7 (43.8%)		
3	1 (1.7%)	0	1 (5.6%)			0	0		
Number of vascular folds between stiches, median (IQR)	0 (0,1)	0 (0,1)	0 (0,1)	1.554 [0.471–5.121]	0.443	0 (0,0)	0 (0,1)	0.115 [0.012–1.129]	0.054
Effectiveness									
Anastomotic vascular patency lasts 5 min	46 (76.7%)	10 (83.3%)	13 (72.2%)	1.923 [0.307–12.053]	0.669	11 (78.6%)	12 (75.0%)	1.222 [0.222–6.730]	1.000
Anastomotic vascular patency lasts 30 min	28 (46.7%)	6 (50.0%)	7 (38.9%)	1.571 [0.359–6.875]	0.547	8 (57.1%)	7 (43.8%)	1.714 [0.403–7.292]	0.464

3D, three-dimensional; ORs, Odds ratios; CI, confidence interval; NA, not applicable; IQR, interquartile range.

### Feasibility

Although the targeted vessel was smaller than 1 mm, the procedure could still be completed using the 3D exoscope, as well as conventional microscope. The operational duration included vessel separation (superficial, *P* = 0.409; deep, *P* = 0.163), temporary occlusion (superficial, *P* = 0.874; deep, *P* = 0.614) and total procedure (*P* = 0.534 for superficial, *P* = 0.681 for deep) durations, which were comparable between conventional microscopes and 3D exoscopes. Blood leakage at the sutures also exhibited no significant difference between the microscope and 3D exoscope (superficial, *P* = 0.466; deep, *P* = 0.389). Furthermore, vascular folds evaluated with harvested vessels were almost not visible with conventional microscopes and 3D exoscopes (superficial, *P* = 0.443; deep, *P* = 0.054) (Table 2).

### Effectiveness

**Figure 2:**
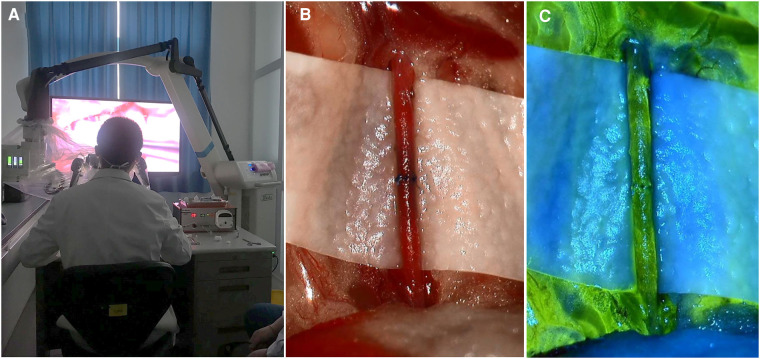
Real scene with 3D exooscope. (**A**) Real scene of operator anastomosis using a 3D exoscope. (**B**) Real situation of anastomotic blood vessel using a 3D exoscope. (**C**) Sodium fluorescein video-angiography for patent anastomotic vessels using a 3D exoscope.

**Figure 3:**
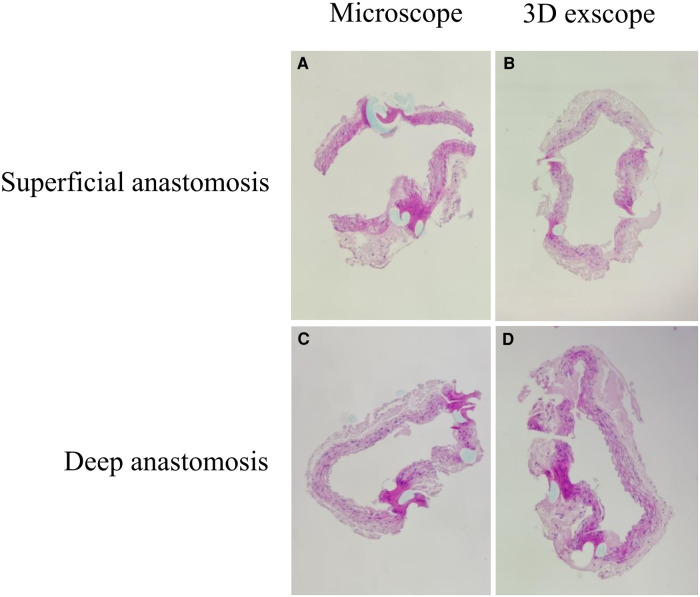
Typical HE staining results (power of magnification: 100×). (**A**) Superficial anastomosis using a conventional microscope. (**B**) Superficial anastomosis using a 3D exoscope. (**C**) Deep anastomosis using a conventional microscope. (**D**) Deep anastomosis using a 3D exoscope.

Our team also evaluated the patency of the anastomotic vessels. We found no difference in patency duration, regardless of the type of microscope used ([5 min] superficial: *P* = 0.669, deep: *P* = 1.000; [30 min] superficial: *P* = 0.547, deep: *P* = 0.464) (Table 2). Given that the 3D exoscope could offer video-angiography, we show vascular patency sodium fluorescein angiography images in Figure 2. Additionally, the results of HE staining also showed comparable outcomes with these two different microscopes (Figure 3).

## Discussion

With the development of neurosurgery, many corresponding techniques and devices were created, such as neuronavigation, ultrasound, intraoperative magnetic resonance imaging (MRI) and/or computed tomography (CT), robotic technology, augmented reality, awake surgery (11). The advent of exoscopes in recent years may bring forth a new era of neurosurgery. At present, many publications have reported the application of exoscopes in the field of neurosurgery (3, 11), most of which focused on brain and spinal surgery; however, some research concentrated on neurovascular bypass. Hafez et al. (6, 7) reported a comparative and laboratory series with the use of exoscopes and conventional microscopes using chicken wing vessels. Nossek et al. (2) reported five patients who underwent bypass surgery with successful revascularization and no exoscope-related complications. The number of patients was too small to demonstrate the reliability of exoscopes in bypass surgery; therefore, it was necessary to perform vascular anastomosis with exoscopes on live animals to further clarify its value.

In this study, we compared the feasibility and effectiveness of a conventional microscope and 3D exoscope for performing microvascular anastomosis *via in vivo* animal experiments; a total of 60 mice were included for analysis. To better adapt to the complexity of clinical practice, we set up two scenes according to the depth of anastomosis to simulate more complicated situations; both visualization tools could complete these operations. For superficial anastomosis, the duration of the operation did not vary for the different microscopes, and when in the more difficult clinical scene of deep anastomosis, using either microscope again did not demonstrate a significant difference. These results indicate that the 3D exoscope had good operability in simple, as well as complex situations. The results of HE staining also demonstrated that both microscopes were able to complete difficult anastomosis well; additionally, using the 3D exoscope was equal to using a conventional microscope for retaining patency in the blood vessels for a certain period of time. As shown in Figure 2, when sodium fluorescein is injected intravenously, the exoscope can clearly demonstrate the patency of the blood vessels; we believe that this will provide great convenience in clinical practice. These results gave us confidence that the use of 3D exoscopes in the future can guarantee successful surgeries.

Two years ago, De Virgilio et al. (12) successfully performed free flap microvascular anastomosis in head and neck connections using 4 K 3D exoscopes in a series of 10 cases. They found that the 3D exoscope could be used to manage anastomoses in vessels as small as 1 mm in diameter. In fact, in 2009, De Virgilio et al. and Ichikawa et al. demonstrated the feasibility of free flap microvascular anastomosis using 3D exoscopes (13, 14). However, there are few reports regarding microvascular anastomosis in the neurosurgical field. We expect that our experimental results would lead to more confidence regarding neurosurgical vascular anastomosis.

Compared with the conventional microscope, the 3D exoscope provided better ergonomics. In a neck-neutral sitting position, the operator could attain more unconstrained movements and a more relaxed posture. Additionally, sharing the 3D view with all visitors made the exoscope an exceptionally good tool for teaching and communicating in laboratory settings. These findings are consistent with those of previous reports (2, 15, 16).

### Limitations

This study has some limitations. First, the sample size was too small, and the results needed to be carefully interpreted. Second, only end-to-end anastomosis was performed to evaluate the effect of using a 3D exoscope, which might have left some issues overlooked. However, there is a scenario where the operation is performed within a 5 cm depth and a diameter limited to 3 cm, which is challenging for the surgeon. We think that different scenarios can offer more information to readers. Third, the outcome measures relied on subjective judgments, which might result in bias. Although some exoscopic systems do not have the ability to perform video-angiography, this kind of exoscope is equipped with two video-angiography modes, which can adapt to the needs of different scenes. Forth, the microscope used in the study is more technically limited compared to those used in high-complexity neurosurgery centers, what influences the image quality and ergonomics of the surgeon.

## Conclusions

In a challenging experimental setting, the conventional microscope and 3D exoscope demonstrated comparable outcomes for microvascular anastomosis in the animal experiments. *In vivo* microvascular anastomosis is feasible and effective using the 3D exoscope. However, larger-scale clinical evaluation is necessary to draw definite conclusions regarding possible advantages in real-word practice in the future.

## Data Availability

The original contributions presented in the study are included in the article, further inquiries can be directed to the corresponding author/s.
